# New Appliances and Things Medical

**Published:** 1896-03-07

**Authors:** 


### NEW APPLIANCES AND THINGS MEDICAL.

[We shall be glad to receive, at our Office, 428, Strand, London, W.C., from the manufacturers, specimens of all new preparations and appliances, which may be brought out from time to time.]

### ICHTHYOL PREPARATIONS.

(Cordes, Hermanni, and Co., 20, High Holborn, W.C.)

Ichthyol has during the last five or six years won the confidence of the profession as a valuable drug in skin diseases and in gynæcological practice; it is highly probable that it will prove equally useful in other branches of surgery and medicine. To cope with its many applications the above firm are now adding to their list a number of new preparations more closely adapted to the various demands that may be made upon it. Samples of the following preparations have been forwarded to us: Alcoholic ethereal lotion of 30 per cent. ichthyol, alcoholic ethereal lotion of 10 per cent. ichthyol, ichthyol wadding (20 per cent.), ichthyol pills, ichthyol capsules, superfatted ichthyol soap, ichthyol toilet soap, sodium ichthyol (pure), ammonium ichthyol (pure), ichthyol plasters, ichthyol paste. It is impossible in a short notice to detail the various applications of the different preparations. The firm, however, publish, in German, a valuable little treatise, "Das Ichthyol," in which 300 receipts are given for all the conditions for which ichthyol has been found of use. We would, however, draw attention to ichthyol wadding and ichthyol paste. The former constitutes a most valuable surgical dressing, and the latter, when painted on indolent ulcers or chronic eczema, forms a protective coating, as well as a medicament of acknowledged merit. Among the newer applications of ichthyol may be mentioned the treatment of rheumatism, neuralgia, gout, chronic catarrhal conditions of the different mucous membranes, the specific exantheurata, syphilis, and gonorrhœa.

### LIQUOR AURI BROMARSENALIS AND RUPEOL.

(Arthur and Co., 74, Newman Street, W.)

We have received two new medicinal preparations from that excellent chemist, Mr. Arthur. The first of them is a combination of bromide of gold and oxy-bromide of arsenic ($Au\ Br_3$, $AsOBr_2$). The bromide of gold is stated to have a controlling influence in conditions of raised excitability of the psychomotor centres, and for this purpose to be superior to the potassium salt; and, combined with the oxy-bromide of arsenic, it has been recommended for epilepsy and allied neuroses. From the point of view of pharmacy it has very decided merits. Its therapeutic value has still to be established; it appears, however, to constitute a hopeful subject for investigation and research. The second preparation, Rupeol, is a petroleum oil of great purity and high boiling point, and made expressly by Mr. Arthur for internal use. It is almost colourless, and, as we can vouch for ourselves, absolutely tasteless. We believe it to be a first-class preparation, and those who advocate the use of petroleums for internal administration will do well to make themselves acquainted with Mr. Arthur's new product.

